# Microstructure and Mechanical Properties of Al-12Si Alloys Fabricated by Ultrasonic-Assisted Laser Metal Deposition

**DOI:** 10.3390/ma13010126

**Published:** 2019-12-26

**Authors:** Yang Zhang, Yuqi Guo, Yan Chen, Yabin Cao, Haibo Qi, Shaopu Yang

**Affiliations:** 1 School of Materials Science and Engineering, Shijiazhuang Tiedao University, Shijiazhuang 050043, China; 2 State Key Laboratory of Mechanical Behavior and System Safety of Traffic Engineering Structures, Shijiazhuang Tiedao University, Shijiazhuang 050043, China

**Keywords:** laser metal deposition, Al-12Si alloy, ultrasonic vibration, remelting treatment

## Abstract

This paper presents a method of ultrasonic-assisted laser metal deposition of Al-12Si alloy. The effects of the ultrasonic power and remelting treatment on the development of porosity, microstructural evolution, and tensile properties of the deposits were investigated. The results suggested that a combination of an ultrasonic vibration and remelting treatment could prolong the existence of the molten pool and the effect of the ultrasound. Therefore, the density of the samples increased from 95.4% to 99.1% compared to the as-prepared samples. The ultrasonic action in the molten pool could not only increase the density of the samples but also refine the grains and improve the tensile properties of the samples. Metallographic observation showed that the maximum size of the primary α-Al dendrites were refined from 277.5 µm to 87.5 µm. The ultimate tensile strength and elongation of the remelting treatment samples with ultrasonic vibration were ~227 ± 3 MPa and 12.2% ± 1.4%, respectively, which were approximately 1.17 and 1.53 times those of the as-prepared samples, respectively. According to the tensile properties and fracture analysis, the density increase dominated the improvement of the mechanical properties.

## 1. Introduction

Al-Si alloys with high specific strength, excellent corrosion, and wear resistance are widely used in the transportation and aerospace fields [1,2]. Traditional methods, such as casting, forging, and extrusion, require molds for the fabrication of aluminum alloy components, leading to high costs for complex-shaped parts with low batch production. Additive manufacturing (AM) technology has near-net-shape capabilities, making this method ideal for the production of complex geometries, effectively solving the problems of the high cost of manufacturing molds and long development cycles [3,4,5].

Laser metal deposition (LMD) is a powder feeding laser additive manufacturing technology that can directly form or repair solid freeform components from computer aided design model files [6,7,8]. During the LMD process, the metal powders are fed by the synchronous powder feeding method. The laser irradiates the metal powder and the surface layer of the substrate to form a molten pool, creating a solid layer after the solidification of the molten pool. The shape of the required part is formed by the repetition of multiple layers. Compared with the selective laser melting (SLM) process, LMD has higher deposition efficiency and is suitable for preparing larger-sized structures [9]. In addition, this method can be conveniently combined with other processing technology such as friction stir process [10].

Since porosity greatly influences the mechanical properties of parts, the first purpose of laser additive manufacturing technology is to obtain a dense structure [11]. Rao et al. [12] obtained a high density AlSi10Mg alloy using the SLM with an optimal energy density range of 50–120 J/mm^3^. Aboulkhair et al. [13] studied the effect of the scan strategy on the elimination of the porosity and concluded that a relative density of 99.8% can be attained by employing a suitable strategy. Dai et al. [14] found that the residual pores of Al-12Si alloys subjected to SLM can be eliminated by remelting the solidified layer. In our preliminary work [15], we found that the application of an ultrasonic vibration during laser metal deposition was an effective measure to increase the density of specimens. Xu et al. [16] also confirmed that ultrasonic vibration was very effective in removing the hydrogen in casting melts of A356 alloys under three different test conditions.

Applying ultrasonic vibration in the casting and welding processes of aluminum alloy can not only remove pores but also refine the crystal microstructure of the metals [17,18,19]. Srivastava et al. [20] analyzed the effects of the ultrasonic intensity on the microstructure of Al-Si, Al-Cu, and Al-Ni binary alloys, the grain sizes of all these aluminum alloys significantly decreased with increasing ultrasonic density. Wang et al. [21] reported the mechanism of the grain refinement in ultrasonic casting of Al-8%Si hypoeutectic alloy. Dinda et al. [22,23] reported that coarse columnar dendrites grow at the boundaries of the molten pools of Al-Si alloys prepared by LMD. Certain studies show that the formation of fine equiaxed grains guarantees better mechanical properties and fewer defects in the deposited metal [24,25]. However, compared with the traditional casting technology of aluminum alloys, very few relevant studies could be achieved by integrating ultrasound into laser additive manufacturing process. Laser processing has rapid heating and cooling characteristics, and the mechanism of ultrasonic action on the short-time laser molten pool is still unclear. Therefore, it is necessary to study the action of ultrasonic vibration in the process of LMD.

In this study, an ultrasonic-assisted laser metal deposition technique has been successfully developed to prepare of Al-12Si alloys. Based on the optimization of the process parameters in our previous study, the effects of ultrasonic action and remelting treatment on the density, microstructure evolution and tensile properties of the Al-12Si alloy were investigated in detail. The mechanism of the pore removal and grain refinement in the short-time laser molten pool of the Al-Si alloy under ultrasonic vibration were explored. The density-microstructure-property relationships in the laser deposited samples were also analyzed.

## 2. Materials and Methods

The substrate material used in the experiment was pure Al (Titd Metal Materials Co., Ltd., Changsha, China) with dimensions of 200 mm × 200 mm × 10 mm. The surface oxide film was removed by alkali and acid washing prior to the test. The Al-12Si alloy powder was produced by gas atomization and had a size distribution range of 60–150 μm and an average particle size of approximately 90 μm. The Al-12Si alloy powder was provided by Titd Metal Materials Co., Ltd. (Changsha, China). Table 1 shows the nominal composition of the powder for the LMD process. The metal powder was dried in a vacuum chamber at 120 °C for 6 h before laser metal deposition.

**Figure 1:**
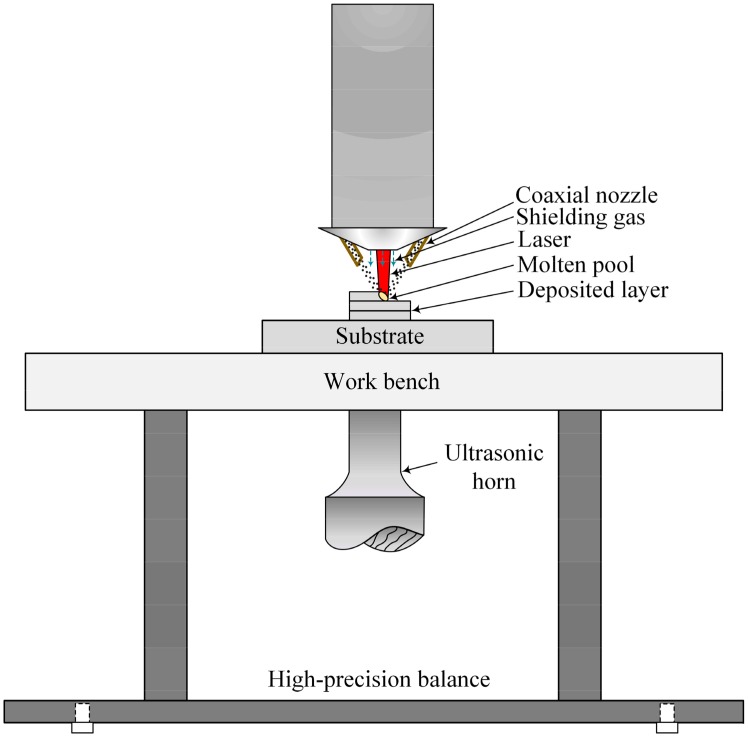
Schematic of ultrasonic-assisted laser metal deposition.

A self-assembly ultrasonic-assisted LMD system was independently developed by Shijiazhuang Tiedao University (Shijiazhuang, China). This system consists of a 4 kW fibre laser unit (YLS-4000) (IPG Photonics Corporation, Webster, MA, USA) with a spot size of 2 mm, a coaxial powder delivery system (DPSF-2) (T.W.T Electrical Industrial Co., Ltd., Xiamen, China), a KUKA Robot (KR30HA-3) (KUKA Roboter GmbH, Augsburg, Germany) and ultrasonic equipment (JY-V8.0, 1kW) (Suzhou Soneek Ultrasonic Technology Co., Ltd., Suzhou, China). To prevent the molten pool from being oxidized, argon gas served as the shielding gas and the powder carrier gas. The flow rates were 15 L/min and 8 L/min, respectively. A schematic diagram of the ultrasonic-assisted LMD system is shown in Figure 1.

**Figure 2:**
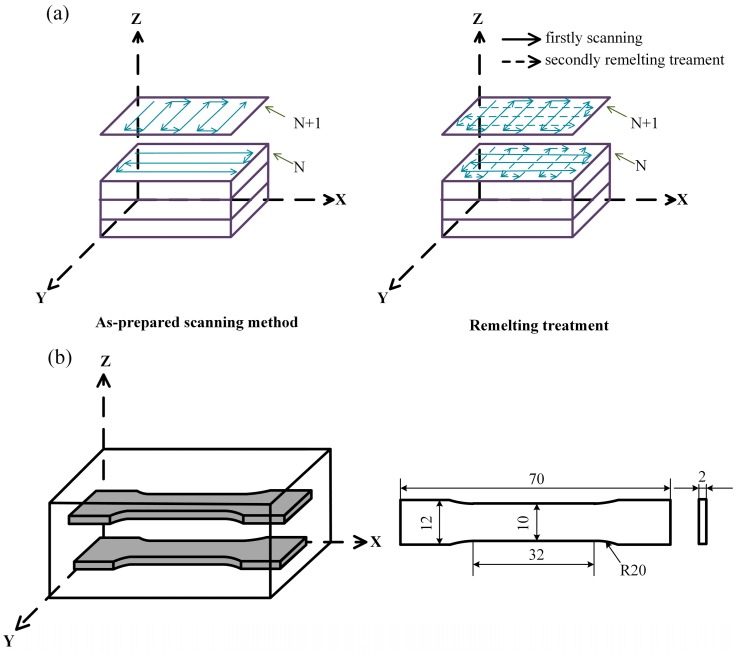
Schematic illustrations showing: (**a**) Deposition methods used to fabricate the samples by laser metal deposition (LMD); (**b**) Tensile test directions and dimensions of the tensile sample.

As shown in Figure 2a, two deposition methods were used in the present investigation, namely, as-prepared scanning (AS) and remelting treatment (RM). In the AS, the laser scanning is continuously preformed during the same layer. All the hatches are parallel to each other, but the scanning directions of two adjacent hatches are opposite. The main difference between the RM and the AS is that the same layer is melted twice in the RM. After the first melting sequence, the remelting direction is perpendicular to the first layer, and no new metal powder is fed. The specimens of the above two scanning methods were rotated 90° between layers to improve the quality of the samples.

During the ultrasonic-assisted LMD process, the laser beam heated the metal powder and the substrate to form a laser molten pool, and ultrasonic vibration was simultaneously introduced from the bottom of the specimen. To obtain denser deposited samples, the optimized fundamental parameters were a laser power of 1100 W, a laser scanning speed of 360 mm/min, a layer thickness of 300 µm, a hatch spacing of 1 mm and a powder flow rate of 1.2 gm/min. The sonotrode was operated at an ultrasonic power of 1000 W, a frequency of 20 kHz, and output amplitude of 25 µm, respectively.

After cutting, the samples were ground with metallographic sandpaper and etched for 10–30 s with a solution of 95 mL H_2_O, 2.5 mL HNO_3_, 1.5 mL HCl, and 1 mL HF. The density of the sample was measured by the Archimedes method. The sample was completely immersed in ethanol, and at least three samples were measured to obtain an average result under each condition. According to the literature results [12], the theoretical density used in the calculations was 2.68 g/cm^3^.

The microstructure of the prepared specimen was observed by optical microscopy (OM) (ZEISS Imager. M2m) (Carl Zeiss Microimaging GmbH, Gottingen, Germany), scanning electron microscopy (SEM) (ZEISS Gemini SEM 300) (Carl Zeiss Microscopy GmbH, Oberkochen, Germany) and electron backscattered diffraction (EBSD) (OXFORD NordlysMax^3^) (OXFORD Instruments, Halifax, UK). The EBSD data were tested at an accelerating voltage of 20 kV with a step size of 6 μm and analyzed using OXFORD Channel5. The axial direction of the tensile specimen was perpendicular to the building direction (Figure 2b). Tensile tests were carried out using the TIANCHEN WOW-10G (China Jinan TIANCHEN Testing Machine Manufacturing CO. LTD, Jinan, China) testing facility with a Fiedler extensometer at room temperature. Three tensile tests were conducted for each condition to obtain average results at a constant strain rate of 0.2 mm/min. The fracture surface morphology was observed by SEM.

## 3. Results and Discussion

### 3.1. Changes in Density of Deposited Samples

**Figure 3:**
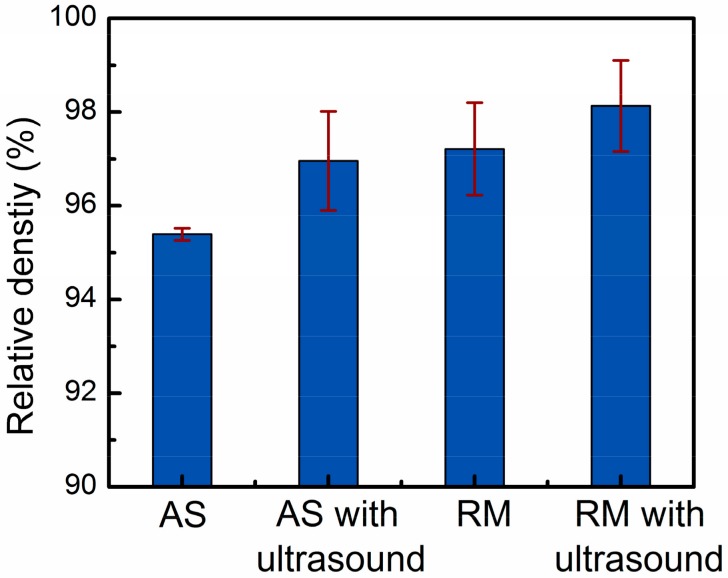
Relative density results of Al-12Si samples prepared by four different parameters.

**Figure 4:**
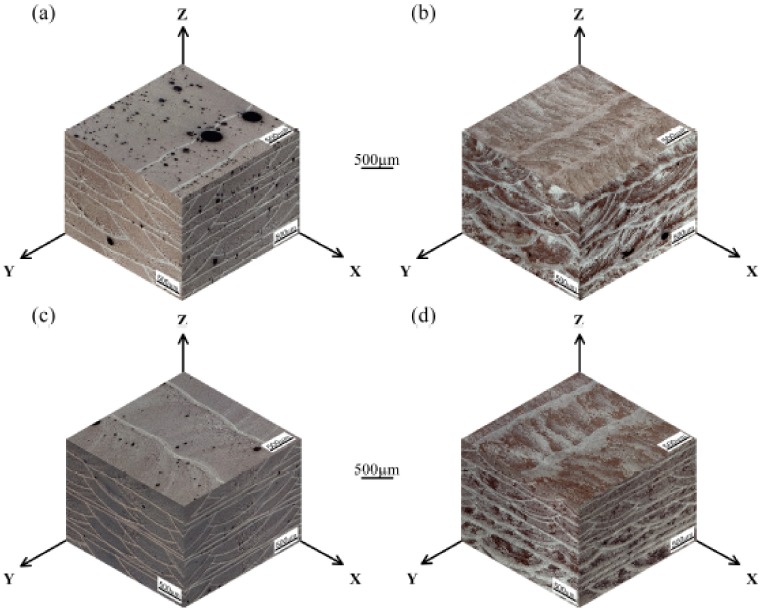
Representative macrostructures of (**a**) as-prepared scanning (AS) sample without ultrasound; (**b**) AS sample with an ultrasonic power of 1000 W; (**c**) Remelting treatment (RM) sample without ultrasound; (**d**) RM sample with an ultrasonic power of 1000 W.

Figure 3 shows the influence of four different parameters on the relative density results of the samples measured by the Archimedes method. As shown in Figure 4, the optical micrographs of the three-dimensional cross-sections produced by four different parameters show the evolution trends of the relative density. The overall trend of the relative density drawn by the histogram is in good agreement with the density change trend of the metallographic photos in Figure 4. For a more detailed analysis, the pore size range, average pore size, number of pores per unit area, and pore volume fraction of the metallographs obtained from four different process conditions were calculated using image analysis and were quantitatively compared in Table 2.

In the as-prepared samples, the average relative density was 95.4% due to the large number of pores in the size range of 3–310 μm (Figure 4a). Under the test conditions of this paper, the dominant type of porosity was dissolved precipitated pores in the interlayer. The metal powders had large specific surface areas, and the crystal water contained in the oxide films on the particle surfaces decomposed into hydrogen by heat [26]. The hydrogen content in the high-temperature molten aluminum was high and suddenly decreased during rapid cooling to precipitate gases [27]. Porosity was generated in the solidified pool when these gas bubbles could not escape the liquid metal surface. Therefore, removing these pores was the key to obtaining dense, high-quality deposited layers.

The application of ultrasonic vibration and remelting treatment were two effective degassing measures, which could increase the average density of the samples to 96.9% and 97.2%, respectively. Table 2 indicates that compared with the AS samples, the samples processed by AS with ultrasonic vibration can obtain higher average pore size, fewer pore size range, number of pores per unit area, and pores volume fraction. With reference of Figure 4b, it can be known that when high-density ultrasonic vibration was applied to a liquid molten pool, some pores escaped from the upper surface of the molten pool, and some pores converged and remained in the deposited layers. To prolong the existence of the molten pool, the deposited layer was remelted (Figure 4c). No new metal powders were fed into the laser molten pool, therefore the remelting process introduced less hydrogen into the molten pool. Moreover, the residual pores in the upper part of the solidified layer could escape by moving to the surface of the molten pool, increasing the specimen density.

As shown in Figure 4d, this paper used a combined approach of ultrasonic degassing and remelting degassing to assist the laser metal deposition of Al-12Si alloy, which integrated the advantages of ultrasonic degassing and remelting degassing. The approach can not only promote the floating and converging of small pores at the bottom of the molten pool, but also extend the existence of the molten pool, which is beneficial to the escape of the pores. Thus, the most compact parts with a density of 99.1% were successfully produced by remelting treatment with an ultrasonic power of 1000 W.

### 3.2. Microstructure Evolution of the Samples

Figure 4 also shows three-dimensional macrostructures of the samples deposited with various processing parameters. The macrostructures exhibited periodic circular and band features in the longitudinal and transverse sections of the deposits due to the circular characteristics of the laser arc and the application of the scanning strategy. The macrostructural nature of the circular or band morphology was essentially the alternation of coarse-grained and fine-grained regions. The circular or band boundaries were represented by bright lines in the optical micrographs. The interesting features of the even layers in the transverse section were analogous to those of the odd layers in the longitudinal section. The morphology in the plan section approximated parallel, horizontal straight lines.

**Figure 5:**
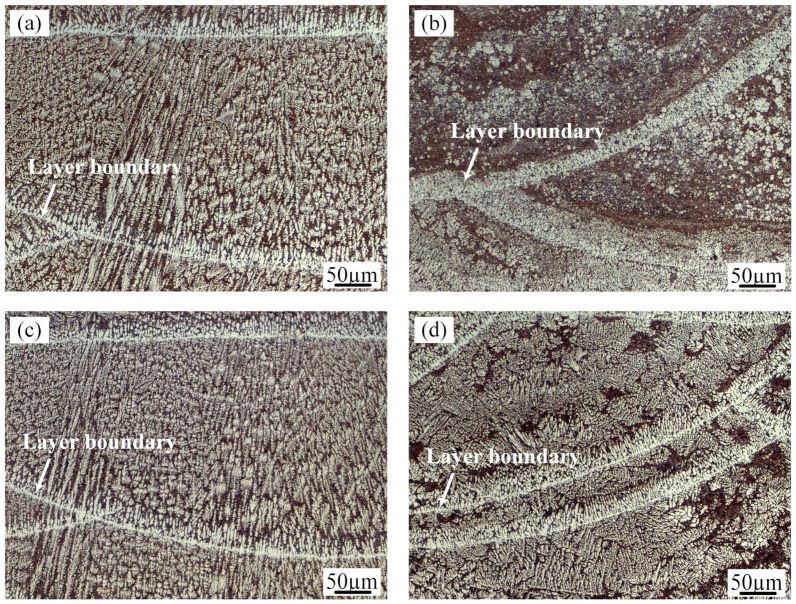
Optical microscopy (OM) micrographs of the Al-12Si samples prepared using (**a**) AS; (**b**) AS under an ultrasonic field; (**c**) RM; (**d**) RM under an ultrasonic field.

Figure 5 shows the optical microstructures of the transverse sections produced by four different process parameters. All of the deposition layers consist of a bright α-Al solid solution and dark Al-Si eutectic. The morphology of the primary α-Al dendrites and their distribution in the Al-Si eutectic structure are significantly affected by processing conditions. During solidification of the specimen produced by the LMD process, nucleation, and growth of new phases occurred when the temperature of the molten poor dropped below the liquidus. The microstructural morphology and grain size of the deposited layer were dependent on the temperature gradient (G), the solidification rate (R), and the cooling rate (G·R) [28].

In the as-prepared samples (Figure 5a), the microstructural characterization varies significantly at different locations in the same molten pool interior. As a result of the high temperature gradient and the low solidification rate at the boundary of the molten pool, the grains are mostly perpendicular to the bead boundary in the form of coarse columnar dendrites. From the boundary to top of the molten pool, with decreasing of the thermal gradient and increasing solidification velocity, the crystalline form of the primary Al translates from columnar dendrites to equiaxed dendrites. Since the crystallization temperature of the Al-Si eutectic is lower than that of the primary Al solid solution, Al-Si eutectic structures are distributed among the α-Al dendrites.

The application of ultrasonic vibration to the deposited layers has little effect on the columnar dendrites at the bead boundary but significantly changes the microstructures of the upper part of the molten pool. With the ultrasonic technique, fine globular Al crystals surrounded by a network of Al-Si eutectic developed in the middle and top of the molten pool in the as-prepared samples, as shown in Figure 5b. The ultrasound treatment causes the fine scale of the microstructure. In addition, the fine Al crystals tend to aggregate in each layer. Figure 5c exhibits the microstructure of the molten pool produced by the remelting treatment without ultrasound. The microstructure is very similar to that of the as-prepared sample. When ultrasonic vibration and remelting are both applied in the molten pool (Figure 5d), the content and size of the α-Al solid solution grains increase compared with those in Figure 5b.

**Figure 6:**
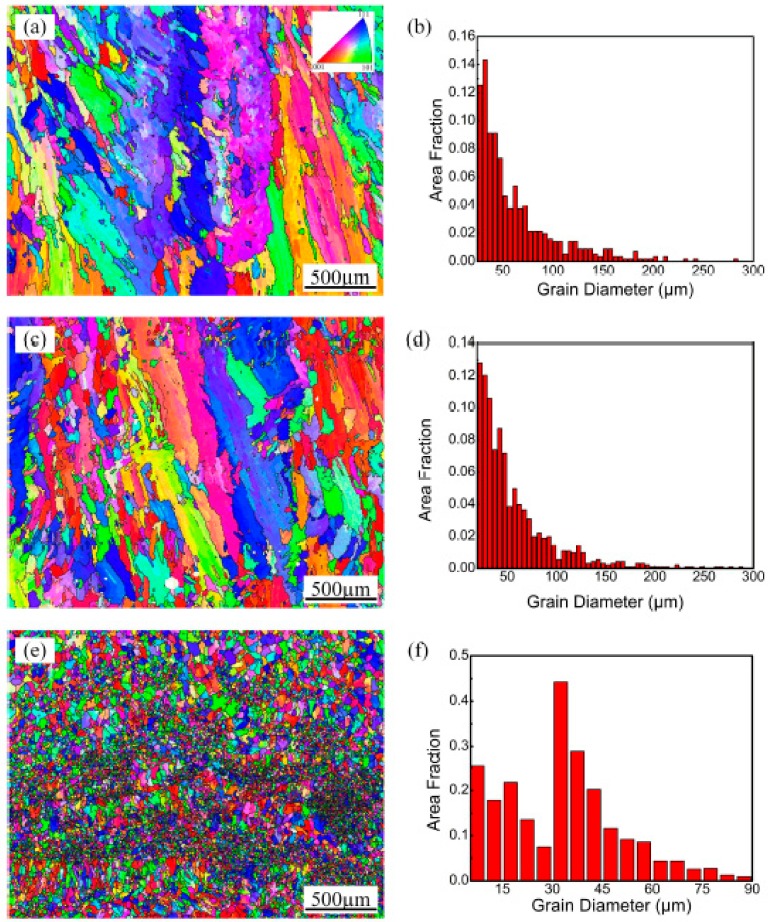
Inverse pole figure (IPF) colored orientation image map and grain size distribution of (**a**,**b**) As-prepared sample; (**c**,**d**) Remelting treatment sample; (**e**,**f**) Remelting treatment sample with ultrasonic vibration.

Figure 6 shows the electron back scattered diffraction (EBSD) results of the transverse sections of the deposited layers made with three different processes. Considering the size and distribution of the Si particles and the EBSD data acquisition step size, the analysis of the morphology and grain size distribution is focused on the Al phase in the present study. Figure 6a,b show that the grain size distribution gradient is large (22.5–277.5 µm) in the as-prepared samples. The morphology of the Al grains in the deposited layer transforms from columnar crystals to equiaxed crystals, but the grain orientation remains unchanged. The height of each deposited layer is approximately 300 μm. The EBSD investigation reveals that there are very large columnar Al grains in each layer, which is almost consistent with the height of the deposited layer.

When the remelting treatment is used individually, as shown in Figure 6c and d, the morphology and size distribution of the α-Al solid solution do not significantly change compared with those of the as-prepared samples. In contrast, the corresponding grain morphology in the remelting treatment samples with ultrasonic vibration is equiaxed with sizes in the range of 7.5–87.5 µm (Figure 6e,f). These fine grains exhibit different colors, implying a randomly distributed grain orientation. This result shows that ultrasonic vibration not only transforms the morphology of the aluminum alloy from a columnar structure to an equiaxed structure but also greatly refines the grain size of the aluminum during the LMD method.

**Figure 7:**
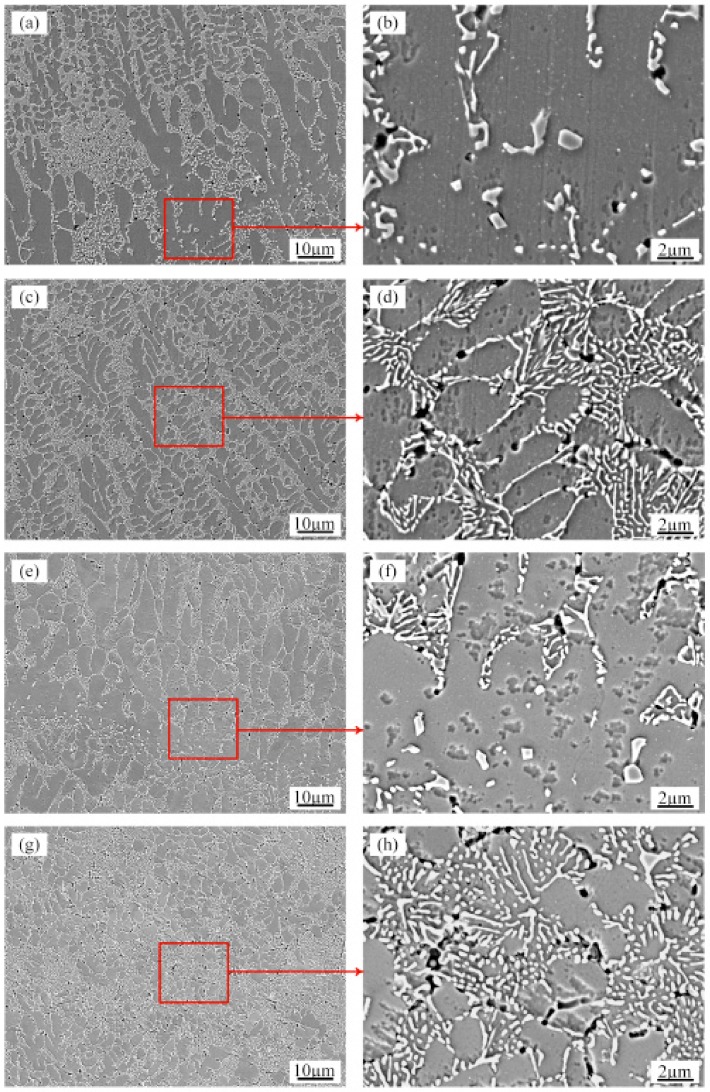
SEM micrographs showing the morphology of Si in the (**a**,**b**) Boundary of the molten pool of the AS samples; (**c**,**d**) Upper part of the molten pool of the AS samples; (**e**,**f**) Boundary of the molten pool of the RM samples with ultrasonic vibration; (**g**,**h**) Upper part of the molten pool of the RM samples with ultrasonic vibration.

Figure 7 shows the typical SEM microstructure of the transverse sections of the as-prepared samples and the RM sample with an ultrasonic power of 1000 W. In all the deposits, the primary aluminum grains are grey features and are decorated with bright white silicon particles. Many small voids, sized approximately 1 μm, are present in the area where the silicon particles are aggregated. These small voids were caused by silicon particles falling off during etching. Comparing the border and upper parts of the SEM images of the molten pool (Figure 7a,c,e,g), the α-Al grains of the RM sample with ultrasonic vibration are significantly smaller than those of the AS sample. SEM observation is carried out to obtain close inspection of the Si particles at different locations in the deposit layer. The microstructural analysis shows no visible influence from the two different processes on the morphology and size of the Si particles in the deposits. Figure 7b and f show that the Si particles at the bottom of the layer boundaries are equiaxed and dispersed with coarse sizes of approximately 1–2 µm due to heat input during laser deposition of subsequent layers. Figure 7d and h show that the sizes of the Si particles at the core of the molten pool are finer than those of the boundaries. The morphology of the Si particles transforms into a fibrous network with sizes smaller than 1 µm.

In general, when ultrasonic waves acted on the Al-12Si alloys produced by laser metal deposition, the main function was to refine the solid solution grains of aluminum. One of the main reasons for the grain refinement during ultrasonic vibration was that cavitation increased the nucleation rate [29]. When the ultrasonic waves were introduced in the laser molten pool from the bottom of the specimen, the cavitation effect, which was produced in the liquid metal, led to the formation of “hot spots” with temperatures of up to 5000 K and a pressure of approximately 1000 atm. Nucleation undercooling could occur from the instantaneous high pressure in the melt, enhancing the nucleation rate [30]. At the same time, the action of the instantaneous high temperature caused the growing dendrites to remelt and inhibited the growth of the crystal grains. Therefore, ultrasonic cavitation improved the nucleation rate and suppressed the growth of the crystal grains, realizing grain refinement.

Another major mechanism of the ultrasonic grain refinement was dendritic fragmentation [29]. During the process of secondary dendrite formation in aluminum alloy melt, cavitation broke down the secondary dendrite arms. These broken dendritic arms were carried by acoustic steaming and dispersed throughout the melt, becoming new nucleation cores, and grain refinement was realized [31,32]. In addition, the acoustic steaming strongly stirred the liquid molten pool under an ultrasonic field, which reduced the temperature gradient and accelerated the cooling rate, making forming small equiaxed crystals easier [33].

### 3.3. Tensile Properties and Fracture Surfaces

**Figure 8:**
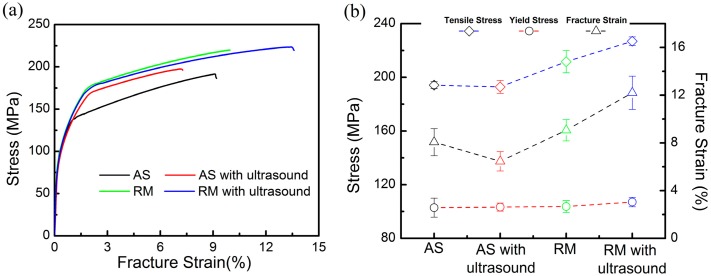
Curves of tensile samples manufactured with the four different processes: (**a**) Tensile stress-strain curves; (**b**) Tensile properties.

Figure 8a records the room temperature tensile stress-strain curves of the samples fabricated with the four different processes. The corresponding yield strength (YS), ultimate tensile strength (UTS) and fracture stain curves are summarized in Figure 8b. The tensile properties of the as-prepared samples reveal a YS of ~102 ± 7 MPa and UTS of ~194 ± 2 MPa with ~8.0% ± 1.1% fracture strain. After 1000 W ultrasonic vibration is applied alone, the tensile properties of the specimen slightly decrease. The combination of ultrasonic vibration and remelting treatment results in an overall trend of improvements in strength and elongation to failure. The YS increases to ~107± 4 MPa and the UTS to ~227 ± 3 MPa along with a plasticity enhancement to approximately 12.2% ± 1.4%, which have approximately 1.17 times the UTS and 1.53 times the fracture strain of the as-prepared samples. The high density and refinement of microstructures in the RM samples with ultrasonic vibration are mainly responsible for this improvement in strength.

**Figure 9:**
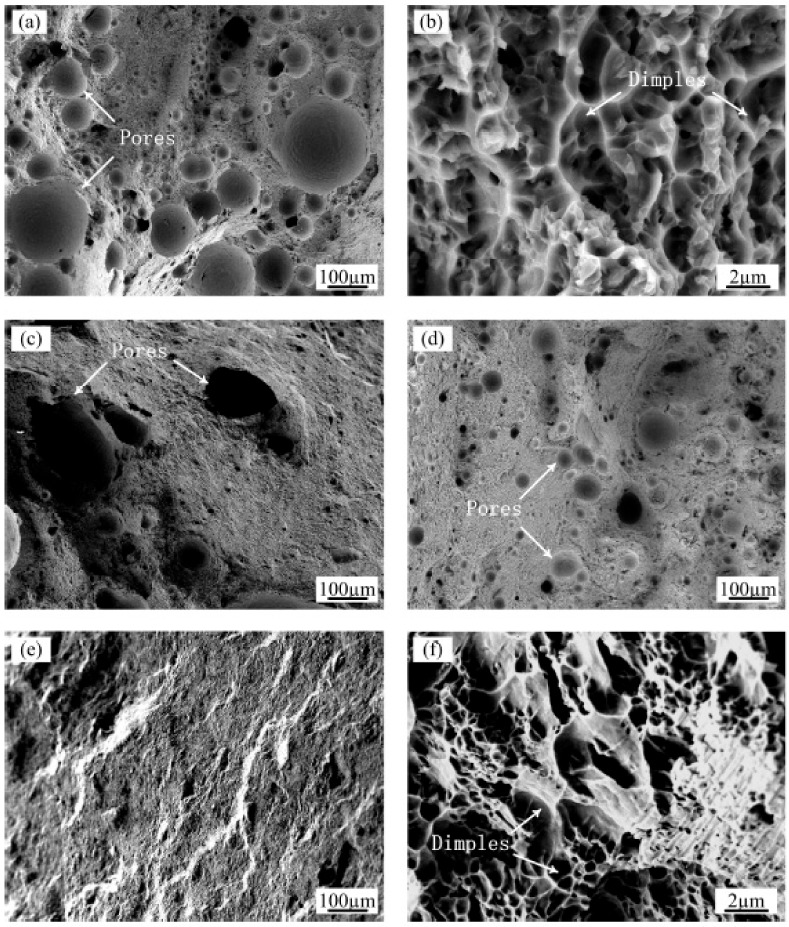
SEM images of the tensile fracture surfaces of (**a**) The AS sample at low magnification and (**b**) high magnification; (**c**) The AS sample with ultrasonic action at low magnification; (**d**) The RM sample at low magnification; (**e**) The RM sample with ultrasonic action at low magnification and (**f**) high magnification.

To explain the evolution of the strength and fracture strain, the surface morphologies of the fracture under four representative process conditions are shown in Figure 9. The as-prepared samples display a high level of porosity with sizes in the range of 8–260 μm on the fracture surface (Figure 9a). At higher magnifications (Figure 9b), small dimples are visible on the surface of the fracture. The fractures tended to initiate from macropores and coalescence with each other, resulting in a torturous crack propagation path. Since the excessive porosity defects degraded the plastic deformation ability and the load carrying capacity, the as-prepared samples had lower ductility and tensile strength.

When using the ultrasonic vibration treatment to assist in the preparation of the aluminum alloy individually, although the deposited layer had the smallest size of the aluminum grains, the mechanical properties were negatively influenced compared with those of the as-prepared samples. This result was mainly due to that the pores converged into irregular shapes (Figure 9c), resulting in failure initiation under lower strains. On the other hand, when the Al-12Si alloys were remelted only, although the microstructure of the samples changed minimally, the tensile properties improved due to the increased density and small size of the residual pores (Figure 9d).

As observed in this study, the highest LMD part densities were obtained by combining the ultrasound and remelting methods. The maximum densities increased from 95.4% to 99.1%, which improved the ultimate tensile strength from 194 MPa to 227 MPa and increased the elongation by a factor of approximately 1.53 times. These phenomena are confirmed by the fractographic analysis of the samples after the tensile tests (Figure 9e,f). From the low magnification views, there are almost no residual pores near the surface of the sample, and the sliding direction of the cracks propagated is approximately 45 degrees to the tensile loading direction. The fracture surfaces of the samples reveal a large number of parabolic shaped dimples, implying perfect ductility during the tensile tests.

As seen from the above analysis, two key factors contributed to the tensile properties, the porosity defects and microstructure refinement formed during the ultrasonic-assisted LMD of the Al-12Si alloys. Between these factors, the influence of the porosity defects on the tensile properties dominates the effect of the grain refinement. The presence of porosity reduced the cross-sectional area, thus weakening the load carrying capacity. In addition, the pores with sharp edges were prone to act as stress concentrators and became a potential source of crack initiation, which decreased the strength and elongation [34].

## 4. Conclusions

In this study, Al-12Si samples were manufactured by laser metal deposition using four different deposition parameters. The evolution of the specimen density, microstructure and tensile properties with ultrasonic and remelting treatments were investigated. The following conclusions were obtained:

(1) The maximum density increased from 95.4% for the as-prepared LMD samples to 99.1% for the samples prepared using the ultrasonic vibration with the remelting treatment. As ultrasonic treatment was applied, small pores were floated, and coalesced to form large pores. Partial remelting of the previous layer prolonged the existence of the molten pool, contributing to the efficient escape of the residual large pores. The combination of the two processes promoted the better densification of the Al-12Si materials during LMD.
(2) The microstructure of the specimens prepared under different processes was composed of the α-Al solid solution and interdendritic Si particles. The evolution of the α-Al solid solution gradually transformed from columnar crystals at the boundary of the molten pool to equiaxed crystals in the upper part of the molten pool. The EBSD measurements showed that the maximum size of the primary α-Al solid solution was significantly refined from 277.5 µm to 87.5 µm due to ultrasonic-enhanced nucleation rate and dendrite fragmentation. The morphology of the Si particles was not homogeneous in each position of a layer. Fibrous Si particles developed in the track cores, and equiaxed Si particles developed at the layer boundary; the ultrasound had negligible effect on the characteristics of the Si particles.
(3) In the LMD process of the Al-Si alloy, the samples fabricated by employing a combination of ultrasound and remelting treatment obtained the highest tensile properties. These samples displayed ultimate tensile strength and fracture strain of approximately 227 MPa and ~12.2%, respectively, which was 17% and 53% higher than the corresponding properties of the as-prepared LMD samples, respectively. The improvement in the tensile properties was mainly attributed to the enhancement of the density and the grain refinement of the LMD parts, wherein density increase dominated the improvement of mechanical properties.

## Figures and Tables

**Table 1:** Chemical composition of the Al-12Si powder material (wt. %).

Alloy Element	Si	Fe	Mg	Zn	Cu	Mn	Al
Al-12Si powder	11.96	0.22	0.0008	0.012	0.0022	0.0006	Balance

**Table 2:** Specified results of the sample pores prepared under different process parameters.

Process Parameters	Pore Size Range (μm)	Average Pore Size (μm)	Number of Pores per Unit Area (N/mm^2^)	Pore Volume Fraction (%)
AS	3–310	10 ± 4	26 ± 12	4.3 ± 1.9
AS with ultrasound	3–150	13 ± 1	10 ± 5	2.8 ± 1.1
RM	3–93	9 ± 3	8 ± 4	2.4 ± 0.9
RM with ultrasound	3–40	6 ± 1	4 ± 2	1.2 ± 0.7
